# Diabetic retinopathy: Looking forward to 2030

**DOI:** 10.3389/fendo.2022.1077669

**Published:** 2023-01-09

**Authors:** Tien-En Tan, Tien Yin Wong

**Affiliations:** ^1^ Singapore Eye Research Institute, Singapore National Eye Centre, Singapore, Singapore; ^2^ Duke-NUS Medical School, Singapore, Singapore; ^3^ Tsinghua Medicine, Tsinghua University, Beijing, China

**Keywords:** diabetic retinopathy, future trends and predictions, epidemiology, pathophysiology, imaging modalities, artificial intelligence, new treatments, classification and staging system

## Abstract

Diabetic retinopathy (DR) is the major ocular complication of diabetes mellitus, and is a problem with significant global health impact. Major advances in diagnostics, technology and treatment have already revolutionized how we manage DR in the early part of the 21^st^ century. For example, the accessibility of imaging with optical coherence tomography, and the development of anti-vascular endothelial growth factor (VEGF) treatment are just some of the landmark developments that have shaped the DR landscape over the last few decades. Yet, there are still more exciting advances being made. Looking forward to 2030, many of these ongoing developments are likely to further transform the field. First, epidemiologic projections show that the global burden of DR is not only increasing, but also shifting from high-income countries towards middle- and low-income areas. Second, better understanding of disease pathophysiology is placing greater emphasis on retinal neural dysfunction and non-vascular aspects of diabetic retinal disease. Third, a wealth of information is becoming available from newer imaging modalities such as widefield imaging systems and optical coherence tomography angiography. Fourth, artificial intelligence for screening, diagnosis and prognostication of DR will become increasingly accessible and important. Fifth, new pharmacologic agents targeting other non-VEGF-driven pathways, and novel therapeutic strategies such as gene therapy are being developed for DR. Finally, the classification system for diabetic retinal disease will need to be continually updated to keep pace with new developments. In this article, we discuss these major trends in DR that we expect to see in 2030 and beyond.

## 1 Introduction

Diabetic retinopathy (DR) is the major ocular complication of diabetes mellitus, and occurs in about 30 to 40% of diabetic individuals (1, 2). Globally, more than 100 million individuals are living with DR, and DR is a leading cause of blindness and visual impairment, especially among the working-age adult population (1, 3). Fortunately, much of the visual loss from DR is preventable, and the rates of vision loss from diabetes and DR have steadily declined over the past few decades (4, 5). Such improvements in visual outcomes for DR are multifactorial, and are due in large part to a combination of better systemic risk factor control, coupled with advances in ocular disease assessment, screening, imaging and treatment in recent years. For example, the universal adoption of DR classification systems such as the Early Treatment of Diabetic Retinopathy Study (ETDRS) and International Classification of Diabetic Retinopathy (ICDR) severity scales that effectively prognosticate the risk of disease progression, coupled with large-scale DR screening programs around the world, have allowed for appropriate surveillance and early intervention to prevent the onset of vision-threatening complications (5–7). Panretinal laser photocoagulation (PRP) helps to prevent severe vision loss due to proliferative DR (PDR), and the introduction of pattern scan laser (PASCAL) has made the procedure quicker, easier to perform, and more comfortable for patients (8–10). The widespread availability and use of non-invasive imaging such as optical coherence tomography (OCT), together with the introduction of intravitreal anti-vascular endothelial growth factor (anti-VEGF) treatments have revolutionized the assessment and treatment of diabetic macular edema (DME), and dramatically improved visual outcomes for this complication of DR (11–13). Surgical outcomes for tractional retinal detachments and diabetic vitrectomies have also improved over the years, with the availability of more advanced instrumentation and surgical adjuncts such as pre-operative anti-VEGF injections (14–16).

Despite the tremendous progress that the field of DR has already seen, there are yet more exciting advances being made. Looking forward over the next decade, many of these ongoing developments are likely to further transform the clinical and research landscapes. In this article, we review some of the recent progress that has been made, and suggest how these developments may continue to shape the field in 2030 and beyond.

## 2 Shifts in epidemiology and disease burden

The global prevalence and disease burden of DR is expected to increase significantly over the next few decades, from about 103 million individuals in 2020, to 130 million in 2030, and 161 million in 2045 (17). Such projections are due to a variety of factors, including the increasing prevalence of diabetes around the world, lifestyle changes, and increasing lifespans and aging global populations (17). This sharp increase in DR disease burden by more than 25% in just 10 years, is likely to further strain healthcare systems and resources that are already stretched. The economic costs associated with DR and its complications are substantial. Direct healthcare costs related to DR in the USA were estimated at $493 million per year in 2004 (18). More recent data is lacking, but it is notable that these estimates were arrived at prior to the introduction of anti-VEGF treatment for DME. Subsequent studies have found that economic costs are significantly higher for patients with DME than without, and much of this is due to the need for costly anti-VEGF treatment (19, 20). Global prevalence of DME is also projected by increase by about 25%, to about 24 million individuals by 2030 (17). The resultant increase in healthcare costs are expected to be staggering.

Perhaps just as important as the overall increase in disease burden, is the projected pattern of increase. Based on epidemiologic projections to 2030, the rates of increase in DR prevalence for traditionally high-income regions such as North America and Europe appear to be relatively low, ranging from 10.8 to 18.0%. In contrast, the rates of increase in middle- and low-income regions such as the Western Pacific (WP), South and Central America, Asia, Africa, the Middle East and North Africa (MENA) are much higher, ranging from 20.6% to as high as 47.2%. In absolute terms, the largest increases by far are projected to occur in MENA, and WP, where the numbers of individuals with DR are expected to rise by more than 6 million in each region respectively (17). This geographic shift in disease burden towards Asia, Africa and WP means that global health strategies to combat DR will need to pivot to follow the shifting disease demographic. Healthcare resources for DR screening, diagnosis, follow-up, and treatment are urgently needed in these areas. Large-scale systematic, rather than opportunistic, DR screening programs that target all patients with diabetes in these regions will allow for early detection and intervention, will be cost-effective, and will reduce rates of vision loss, but they require significant investment in infrastructure and time to set up (21–24).

## 3 Non-vascular aspects of diabetic retinal disease

The clinically-visible retinal lesions associated with DR, such as microaneurysms, hemorrhages and hard exudates, are primarily the result of retinal microvascular damage. Consequently, the focus on DR pathophysiology, diagnosis and assessment has traditionally always centered around the vascular aspect of the disease. However, with the availability of better structural retinal imaging modalities and functional assessments, evidence has accumulated over the years of significant retinal neural dysfunction as well, which occurs together with, or in some cases precedes, the development of vascular abnormalities. These structural and functional changes have collectively been termed “diabetic retinal neurodegeneration” (DRN) (25–28).

OCT studies have shown that patients with diabetes demonstrate significant thinning of the inner retinal layers, including the retinal nerve fiber layer (RNFL), and ganglion cell layer (GCL) (26, 29–31). Retinal thinning is progressive over time, and can precede the development of clinically-visible DR lesions (26, 30). Histological studies on enucleated eyes also corroborate these findings, showing reductions in retinal ganglion cell density in eyes with DR (32). Functional assessments in diabetes reveal reductions in contrast sensitivity, visual field defects, electrophysiologic deficits, and impaired pupillary responses (33–38).

Despite the clear evidence of DRN occurring in diabetic retinal disease, there remain many important unanswered questions in this area. What is the prognostic significance of DRN in terms of ocular or systemic outcomes in diabetes? What is the functional impact of DRN on quality of life? How and when should DRN be assessed and quantified? Current OCT studies on DRN measure different retinal layers (e.g. RNFL, GCL), and in different, non-standardized locations. Functional assessments such as electrophysiology, visual field perimetry and pupillometry are often time-consuming and resource-intensive. Recently, a portable, handheld chromatic pupillometer was shown to able to provide rapid, clinic-based assessment of retinal neural function in diabetes (38). Such findings, however, need to be replicated and validated in larger cohorts. There is also much ongoing work to determine the prognostic impact of DRN, and to incorporate such assessments of DRN into routine DR classification and staging systems (28, 39, 40). These efforts are likely to change the way we routinely assess and manage DR in the next few decades.

## 4 New imaging modalities and biomarkers

New imaging modalities such as ultra-widefield (UWF) retinal imaging and OCT angiography (OCTA) have been available for research and commercial clinical use for a number of years now. UWF retinal imaging provides a field of view of about 110° to 220°, and allows for visualization up to at least the anterior edge of the ampullae of the vortex veins (41). These platforms can be used for UWF color or pseudocolor photography (UWFCP), as well as UWF fluorescein angiography (UWFFA). UWF imaging platforms are non-contact and often do not require pupillary mydriasis, but their most important advantage, is that they provide for assessment of the retinal peripheries, and overall a much larger retinal surface area than standard color fundus photography (CFP). With standard CFP, the typical 7 standard ETDRS fields cover only about 30% of total retinal surface area (39, 42). In contrast, UWF imaging systems allow for assessment of approximately 80% of retinal surface area, which is a major advantage (42).

Assessment of the retinal peripheries with UWF images in DR has significant prognostic and management implications. For one, inclusion of the peripheries in UWFCP images results in a greater DR severity level in 10 to 19% of eyes (43–46). Furthermore, studies from a longitudinal cohort showed that various peripheral DR lesions, such as predominantly peripheral lesions (PPLs), and number, surface area, and distance of hemorrhages/microaneurysms and cotton-wool spots from the optic nerve head, were independently associated with greater risk of progression to PDR (47, 48). However, the prospective longitudinal Diabetic Retinopathy Clinical Research Network (DRCR.net) Protocol AA study recently concluded that PPLs in UWFCP images were not correlated with DR worsening, whereas PPLs and non-perfusion on UWFFA were (49, 50). Unfortunately, UWFFA has some major drawbacks that limit its universal use in all DR patients, including the need for invasive dye administration, time needed for acquisition, and the need for tertiary specialist interpretation. At present, the ideal modality for peripheral assessment and the best way to do so in DR remain unclear. Nevertheless, it is clear that as we better define the role of the retinal peripheries, UWF imaging platforms are sure to play an important role in DR assessment and management over the next decade.

OCTA is another imaging platform that will be increasingly important in DR assessment and prognostication. OCTA is a non-invasive, non-contact system that can provide angiographic information without the need for invasive dye administration like fluorescein. Other advantages of OCTA over dye-based fluorescein angiography are better visualization of the capillary microvasculature, and depth-resolved segmentation of the superficial, middle and deep capillaries plexuses, which are differentially affected in diabetes and DR (51–53). OCTA can provide quantitative metrics relating to the retinal microvasculature, and many of these, such as lower vessel density, lower fractal dimension, greater tortuosity, and greater foveal avascular zone area, have been associated in cross-sectional studies with greater DR severity (51–55). The impact of such cross-sectional associations in clinical practice is limited, but the major impact from OCTA will be realized when such OCTA metrics are eventually linked to clinical outcomes of interest on longitudinal studies. At present, longitudinal prospective OCTA studies are limited, but hopefully this need will be addressed in the next few years (56–59). Other barriers to widespread adoption and clinical impact of OCTA include scan quality and gradability, as well as the use of multiple different commercial OCTA machines, with proprietary algorithms and quantitative metrics that are not standardized or interchangeable between devices. As these barriers are addressed, it is likely that OCTA will become a powerful, non-invasive prognostic tool for clinical assessment in DR.

## 5 Artificial intelligence

Artificial intelligence (AI) and deep learning (DL) algorithms will play an increasingly important role over the next decade in the areas of medical diagnostics, screening, prognostication, and assisting with management or treatment decisions. Ophthalmology has been a leader in developing AI algorithms for clinical use, and automated diagnosis or detection of DR from CFP images was one of the first use cases developed, from as early as 2016 (60–62). Initial studies already demonstrated that AI algorithms developed on large datasets could reach very high levels of diagnostic performance for detection of referable DR and vision-threatening DR (61, 62). About 5 years later, there are now multiple AI-based systems for DR screening that have been approved for clinical use. IDx-DR (IDx LLC, Coralville, IA, USA) and EyeArt (Eyenuk, Inc., Woodlands Hills, CA, USA) have both received approval by the USA Food and Drug Administration (FDA), and are already in clinical use (63, 64). SELENA+ (EyRIS Pte Ltd, Singapore) has received European CE Mark Approval, and is planned to be deployed as part of the national DR screening program in Singapore soon. An economic modelling study suggested that incorporation of such an AI algorithm as an assistive tool in a large scale DR screening program will be associated with significant cost savings (65). It is likely that by 2030, we will see AI algorithms routinely deployed in many large-scale DR screening programs around the world, either as fully autonomous systems, or in hybrid systems where the algorithms function as assistive tools (65). However, there are still some challenges that need to be overcome for widespread acceptance of large-scale AI screening systems. Retinal images frequently contain signs of other ocular or systemic diseases besides DR, and the medicolegal aspects of this are still uncertain. IDx-DR, for example, only detects DR, and the FDA approval for its use clearly states that the algorithm does not diagnose any other ocular disease. Other AI-based systems take a different approach to this; SELENA+ detects DR, as well as 2 other major eye diseases – age-related macular degeneration and glaucoma (62). Poor image quality can also adversely affect the accuracy of such algorithms, but most commercial AI systems now have in-built automated image quality assessments (62, 63).

Beyond just diagnosis and screening of DR, there are other potential use cases for AI algorithms that are also being developed. AI-based detection of DME from CFP images is promising, and could help to improve and reduce false positive referral rates from DR screening programs (66). Some imaging modalities such as OCT and OCTA have in-built software and segmentation algorithms that provide quantitative parameters, such as central subfield thickness (CST) in OCT, or capillary vessel density in OCTA. However, the capability of these automated software algorithms to provide detailed quantitative information is limited to a few parameters, and is dependent on the accuracy and resolution of automated segmentation. Using AI to improve retinal layer segmentation and to provide precise quantification of fluid volumes in different fluid compartments could have major impact in terms of prognostication, and guiding treatment decisions for DME (67–71). Similarly, there has been a shift in emphasis towards quantitative assessment in modalities that are typically assessed qualitatively or categorically, such as number, size and location of retinal vascular lesions on CFP or UWFCP images, or areas of retinal non-perfusion on UWFFA images (48, 50, 72–74). Manual grading and assessment of these quantitative parameters would be impractical, and AI algorithms for automated quantification will go a long way to making such quantitative parameters accessible, and clinically useful. Finally, the use of AI to process multimodal clinical and imaging data in DR, to provide more accurate prognostication of long-term outcomes, such as visual outcomes, risk of developing incident DME, and anti-VEGF treatment burden in DME, is an exciting area to look forward to (75).

## 6 New treatment strategies

Intravitreal anti-VEGF therapy is the established first line treatment for center-involved DME, and has also been shown to be a valid treatment option for PDR (12, 76, 77). Observations from the registration trials for anti-VEGF therapy in DME showed that anti-VEGF therapy can also result in significant improvements in DR severity for patients with non-proliferative DR, and this has been confirmed in more recent prospective clinical trials as well (78–81). As a result, intravitreal aflibercept is now FDA-approved for treatment of non-proliferative DR, as well as PDR and DME. However, at this point, it seems unlikely that anti-VEGF therapy will be used on a large scale for routine treatment of non-proliferative DR. The DRCR.net Protocol W trial showed that anti-VEGF therapy for non-proliferative DR could prevent the onset of PDR and DME, but that final visual outcomes were no different from a strategy of initial observation, with treatment for PDR or DME initiated as-needed (81). Furthermore, while anti-VEGF therapy results in regression of vascular lesions and apparent “improvement” in DR severity, reports show that the underlying retinal ischemia is unchanged, and that lesions and retinopathy often recur rapidly after cessation (82, 83). Finally, the cost-effectiveness of treating non-proliferative DR with regular anti-VEGF therapy has not been well-examined, but it is difficult to imagine widespread use outside of high-resource clinical settings.

Instead, new treatments that are more likely to have significant impact on the DR landscape over the next decade are those targeting new pathophysiologic pathways, and those that improve the durability of treatment effect. For example, faricimab is a bi-specific monoclonal antibody that provides dual inhibition of both the VEGF and the angiopoietin (Ang) and tyrosine kinase with immunoglobulin-like and epidermal growth factor homology domains (Tie) pathways (84, 85). Inhibiting Ang-2 on top of VEGF-A is thought to provide a synergistic effect, with better vascular stability and reduction in vascular leakage (84). The recent phase 3 YOSEMITE and RHINE clinical trials demonstrated that intravitreal faricimab for DME provided substantial visual gains comparable to aflibercept, but with superior anatomic outcomes. More importantly, faricimab had a durable treatment effect, with more than 70% and 50% of eyes reaching dosing intervals of every 12 to 16 weeks, and 16 weeks respectively at 1 year (85). Other promising treatment strategies to provide increased durability and reduced treatment burden include high-dose aflibercept (8 mg), sustained delivery of ranibizumab through a refillable port delivery system (PDS), and gene therapy with agents such as RGX-314 and ADVM-022 for long-term VEGF suppression (86–89). By providing more durable treatment effect, these approaches aim to address real unmet needs in DME treatment, where high treatment burden, problems with compliance to therapy, and under-treatment limit real world visual outcomes (90–93). These treatment approaches will play a major role in DME management in the near future.

## 7 An updated classification system for diabetic retinal disease

As a consequence of these many exciting advances in the field of DR over the past few decades, our DR classification and severity staging systems need to be updated to keep pace with the latest developments (39, 40, 94). The ETDRS and ICDR severity scales that are in routine use have made tremendous impact to research trials and clinical management, but they are now 2 to 3 decades old, and have significant limitations (7, 95). Some of the key areas that need to be addressed in an updated classification system are: (1) Inclusion of relevant prognostic information from the retinal peripheries that can now be reliably imaged with UWF systems, (2) Recognition and assessment of non-vascular aspects of diabetic retinal disease, such as retinal neural dysfunction or DRN, (3) Incorporating information and biomarkers from available imaging modalities such as OCT and OCTA, (4) Greater emphasis on, and clinically-relevant severity classification for DME, which is now the most common cause of visual impairment from DR, and which drives management decisions, and (5) Accurate prognostication of eyes that have undergone intravitreal anti-VEGF or other treatments.

There are major international efforts ongoing to update the DR classification system, such as the Diabetic Retinal Disease Staging System Update Effort, a project which is part of the Mary Tyler Moore Vision Initiative, which brings together leading scientists and experts on DR, with the overall aim of preventing vision loss from diabetes (94). There are still many gaps and unmet needs in the literature that need to be addressed, to inform a robust, evidence-based updated classification system. Nevertheless, it is likely that we will see a new and improved DR classification and staging system soon, that will have major impact on how we practice and manage DR in 2030. Such a classification system will no doubt need to be validated, regularly reviewed, and further updated to keep pace with new developments in the field. Furthermore, various widely-used international DR management guidelines, such as those by the International Council of Ophthalmology (ICO), will also need to be updated in accordance with new classification systems (76).

## 8 Conclusion

Clearly, many important strides have been made in the field of DR over the past few years, which will shape and transform the clinical and research landscapes in the years to come. Here, we have attempted to anticipate and predict some of these trends that are likely to be influential over the next decade. While many of these new imaging, assessment and treatment modalities have the potential to significantly improve clinical outcomes in DR, it is important that these advances are translated equally to both high- and low-resource settings around the world. As we have discussed above, epidemiologic projections suggest a continued shift towards increased disease burden in low-resource settings, and advances in DR management must be accessible to these patient populations, if we hope to see continued reductions in the rates of visual loss and blindness from DR in 2030 and beyond.

## Author contributions

T-ET and TYW both contributed to conception of the study, and drafting and revising of the manuscript. All authors contributed to the article and approved the submitted version.
